# 123-Iodine MIBG in the Assessment of Sympathetic Denervation in Ogilvie's Syndrome

**DOI:** 10.1055/s-0042-1750387

**Published:** 2022-09-09

**Authors:** Amit Bhoil, Sobhan Vinjamuri

**Affiliations:** 1 Department of Nuclear Medicine, The Royal Liverpool University Hospital NHS Trust, Liverpool, United Kingdom

**Keywords:** I-123 MIBG, autonomic neuropathy, Ogilvie's syndrome, pseudo-obstruction, sympathetic dysfunction

## Abstract

123-Iodine metaiodobenzylguanidine (I-123 MIBG) imaging is frequently used in the assessment of sympathetic innervation and autonomic dysfunction in patients with cardiac failure, neurodegenerative Parkinson's syndrome, multiple system atrophy, myotonic dystrophy, and diabetic mellitus. The etiology of pseudo-obstruction remains unknown with likely imbalance between sympathetic and parasympathetic innervation proposed as a hypothesis. We present a case demonstrating the utility of I-123 MIBG scintigraphy for evaluating a case of pseudo-obstruction requiring frequent hospitalization due to progressive complex autoimmune neurological disorder.

## Case Report

**Fig. 1:**
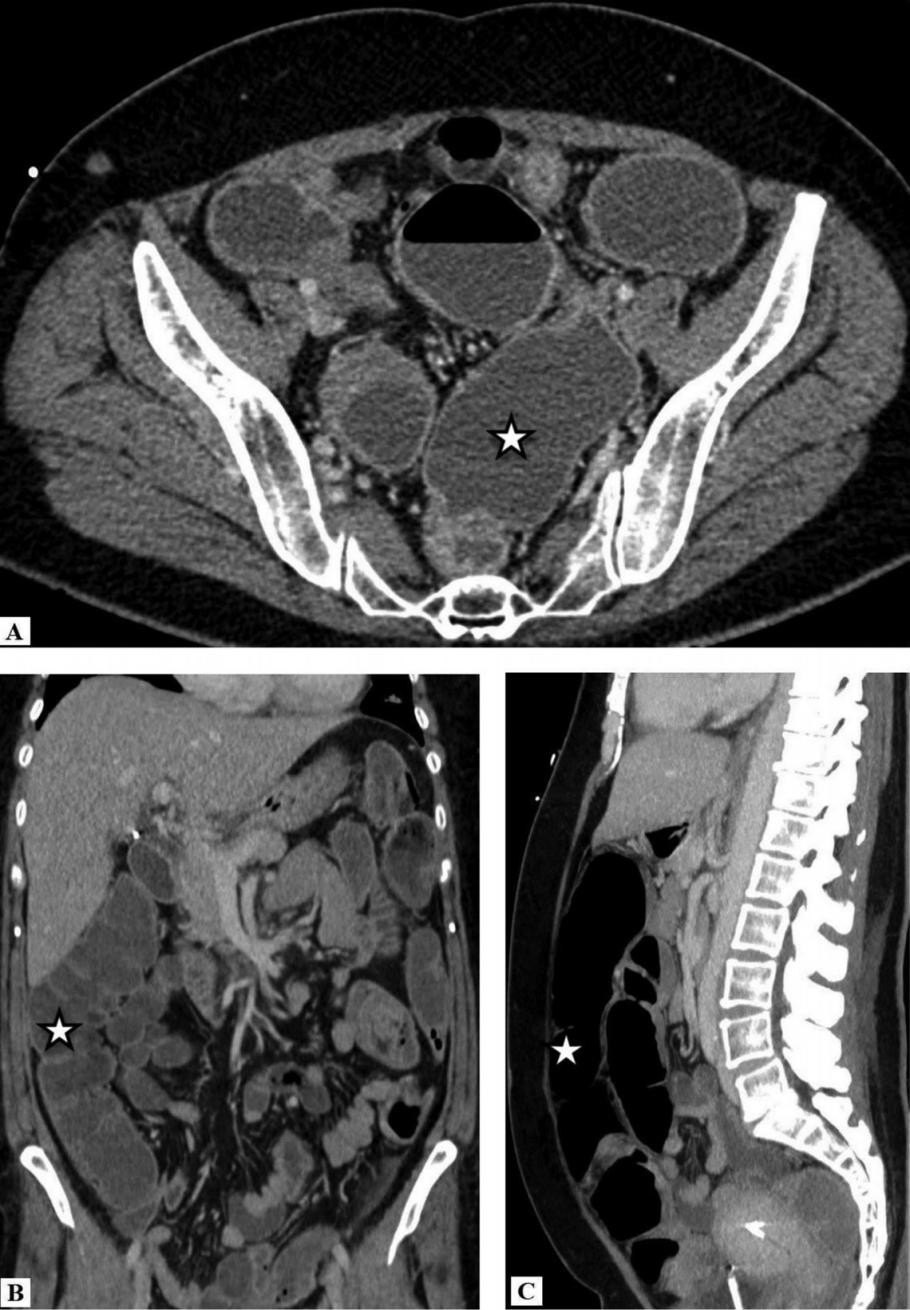
Contrast-enhanced computed tomography (CECT) scan in the ( **A** ) axial, ( **B** ) coronal, and ( **C** ) sagittal planes shows narrowing in the distal sigmoid colon with nonspecific proximal large bowel dilatation.

**Fig. 2:**
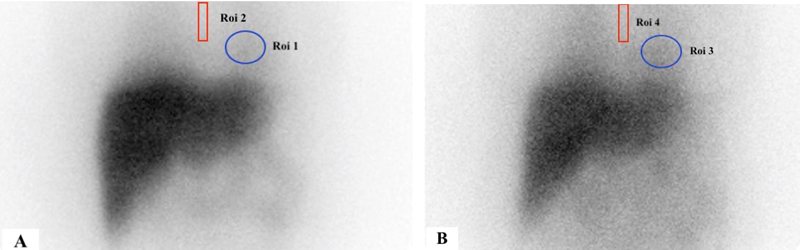
The cardiac ^123^ I MIBG scan with the quantification of heart and mediastinal (H/M) ratio at the early time point of 15 minutes and at the delayed time point of 4 hours showed a reduced (H/M) ratio ( **A** ) at the early time point of 1.58 (control: 2.81) and ( **B** ) at the delayed time point 1.54 (control: 3.04).

A 48-year-female presented with a complex history of chronic back pain with frequent episodes of hospitalization due to pseudo-obstruction with history of dysautonomia for the last 2 years. The contrast-enhanced computerized tomography (CECT) scan in the axial ( Fig. 1A ), coronal ( Fig. 1B ), and sagittal plane ( Fig. 1C ) showed nonspecific large bowel dilatation proximal to the short segment narrowing in the distal sigmoid colon, with no feature of true obstruction. Colonic transit capsule study was normal with no transit delay. The patient progressively had swallowing difficulty, which on video fluoroscopy study was diagnosed with pharyngeal and esophageal phase dysphasia. She later developed unexplained spastic paraplegia with sustained clonus and autonomic pain over time period. The patient had a family history with mother having similar neurological disorder, hence genomic testing was considered. The hereditary spastic paraparesis genomic test for 129 gene and autoimmune autonomic ganglionopathy was negative. The plasma concentration of norepinephrine was within normal limits. Patient was suspected with poorly characterized syndrome of autonomic failure and considered for cardiac 123-iodine metaiodobenzylguanidine (I-123 MIBG) scan for the assessment of the autonomic dysfunction. The cardiac I-123 MIBG scan showed reduced myocardial uptake in the early (15minutes) ( Fig. 2A ) and delayed (4hours) ( Fig. 2B ) images, with the quantification of heart and mediastinal (H/M) ratio at early time point of 15minutes 1.58 (control: 2.81) 1 and at delayed time point of 4hours 1.54 (control: 3.04). 1 The findings were suggestive of cardiac sympathetic denervation. The findings supported the diagnosis of progressive autoimmune autonomic neuropathy and hereditary spastic paraparesis with gastrointestinal and cardiac dysfunction. The patient was symptomatically treated, with nasojejunal feeding and cold octreotide therapy.

## Discussion

Ogilvie's syndrome, or acute colonic pseudo-obstruction (ACPO), is a rare multifactorial disorder that consists of dilatation of part or all of the colon and rectum. The pathophysiology of ACPO is incompletely understood with an imbalance of sympathetic and parasympathetic innervations, being the most widely-postulated theory. However, recently sacral parasympathetic denervation causing atonic distal colonic segment similar to adynamic ileus is suspected as the likely postulated cause. 2 3

The parasympathetic nerve endings release acetylcholine, activating the muscarinic receptors stimulating the plexus activity of entire nervous system, leading to stimulation of bowel movements, gastrointestinal secretion, and blood flow. However, the sympathetic nerve endings release norepinephrine, which inhibits both the plexus of the enteric nervous system through activation of the α _1_ , α _2_ , and β adrenergic receptors. The effects of sympathetic nervous system are further augmented by a presynaptic norepinephrine-mediated inhibition of release of parasympathetic acetylcholine. 4

I-123 MIBG as a radionuclide tracer is an analogue of norepinephrine, and concentrated in adrenergic neurons in the presynaptic vesicles, the concentration reflects scintigraphic display of the adrenergic nervous system. The change in concentration of myocardial sympathetic innervation reflects neuronal integrity and functions. 5 6 The autonomic nervous system abnormalities may be regional, with the adrenergic nerves of the heart particularly vulnerable to the effect of this disease. 6 The scintigraphic display of the adrenergic nervous system with the late H/M ratio is an index of relative distribution of sympathetic nerve terminal offering information about neuronal integrity and function. 7 I-123 MIBG has been reported to provide information regarding cardiac sympathetic function in heart disease, Parkinson's disease, myotonic dystrophy, multiple system atrophy, diabetes mellitus, and Chagas heart disease. 5 8 9 10

## Conclusion

This case demonstrates the potential use of I-123 MIBG scintigraphy for the assessment of the autonomic function of sympathetic denervation with correlation with MIBG uptake in clinical condition as progressive degenerative autoimmune autonomic neuropathy.

## References

[1] Chung E J Lee W Y Yoon W T Kim B J Lee G H MIBG scintigraphy for differentiating Parkinson's disease with autonomic dysfunction from Parkinsonism-predominant multiple system atrophy Mov Disord 2009 24 11 1650 1655 19514077 10.1002/mds.22649

[2] Harnsberger C R Acute colonic pseudo-obstruction (Ogilvie's syndrome) Semin Colon Rectal Surg 2019 30 03 100690

[3] Wells C I O'Grady G Bissett I P Acute colonic pseudo-obstruction: a systematic review of aetiology and mechanisms World J Gastroenterol 2017 23 30 5634 5644 28852322 10.3748/wjg.v23.i30.5634 PMC5558126

[4] Osinga T E Kerstens M N van der Klauw M M Intestinal pseudo-obstruction as a complication of paragangliomas: case report and literature review Neth J Med 2013 71 10 512 517 24394736

[5] EANM Cardiovascular Committee European Council of Nuclear Cardiology Flotats A Carrió I Agostini D Proposal for standardization of 123I-metaiodobenzylguanidine (MIBG) cardiac sympathetic imaging by the EANM Cardiovascular Committee and the European Council of Nuclear Cardiology Eur J Nucl Med Mol Imaging 2010 37 09 1802 1812 20577740 10.1007/s00259-010-1491-4

[6] Sisson J C Shapiro B Meyers L Metaiodobenzylguanidine to map scintigraphically the adrenergic nervous system in man J Nucl Med 1987 28 10 1625 1636 3655915

[7] Wakabayashi T Nakata T Hashimoto A Assessment of underlying etiology and cardiac sympathetic innervation to identify patients at high risk of cardiac death J Nucl Med 2001 42 12 1757 1767 11752070

[8] Genovese E A Mallardo V Cipullo S [Cardiac sympathetic innervation imaging with myocardial MIBG scintigraphy] Recenti Prog Med 2013 104(7-8):356 360 24042407 10.1701/1315.14575

[9] Ichikawa Y Takeda K Murashima S Increased myocardial uptake of iodine-123 metaiodobenzylguanidine on delayed images, compared with early images, in patients with multiple system atrophy Clin Nucl Med 2003 28 11 890 892 14578702 10.1097/01.rlu.0000093283.50688.b2

[10] Landesmann M C da Fonseca L M de B Pereira B Iodine-123 metaiodobenzylguanidine cardiac imaging as a method to detect early sympathetic neuronal dysfunction in chagasic patients with normal or borderline electrocardiogram and preserved ventricular function Clin Nucl Med 2011 36 09 757 761 21825843 10.1097/RLU.0b013e31821772a9

